# Immunization with a Double-Mutant (R192G/L211A) of the Heat-Labile Enterotoxin of *Escherichia coli* Offers Partial Protection against *Campylobacter jejuni* in an Adult Mouse Intestinal Colonization Model

**DOI:** 10.1371/journal.pone.0142090

**Published:** 2015-11-05

**Authors:** M. John Albert, Shilpa Haridas, Mathew Ebenezer, Raj Raghupathy, Islam Khan

**Affiliations:** 1 Departments of Microbiology, Faculty of Medicine, Kuwait University, Jabriya, Kuwait; 2 Department of Biochemistry, Faculty of Medicine, Kuwait University, Jabriya, Kuwait; University of Hyderabad, INDIA

## Abstract

We have previously shown that antibodies to cholera toxin (CT) reacted with the major outer membrane proteins (MOMPs) from *Campylobacter jejuni* strains on Western blot. Further, oral immunization with CT significantly protected against challenge with *C*. *jejuni* in an adult mouse colonization model of infection. CT and the heat-labile enterotoxin (LT) of enterotoxigenic *Escherichia coli* are structurally and functionally related. LT and its mutants including the double-mutant LT (R192G/L211A) (dmLT), are powerful mucosal adjuvants. Unlike LT which is reactogenic, dmLT has been shown to be safe for human use. In the current study, we determined whether rabbit anti-dmLT antibodies reacted with MOMPs from *C*. *jejuni* strains and whether immunization with dmLT would afford protection against *C*. *jejuni*. On Western blot, the MOMPs from *C*. *jejuni* 48 (Penner serotype O:19), *C*. *jejuni* 75 (O:3) and *C*. *jejuni* 111 (O:1,44) were probed with rabbit antibodies to dmLT or LT-E112K (a non-toxic LT mutant), which showed a lack of reaction. Adult BALB/c mice were orally immunized with dmLT and orally challenged with *C*. *jejuni* 48 or 111. Protection from colonization with the challenge bacteria was studied by enumerating *Campylobacter* colonies in feces daily for 9 days. Vaccination produced robust serum and stool antibody responses to dmLT and no antibody responses to *C*. *jejuni* MOMP. Vaccinated mice showed reduced colonization and excretion of both challenge strains compared to control mice. However, the differences were not statistically significant. The protective efficacy of the dmLT vaccine varied from 9.1% to 54.5%. The lack of cross-reaction between the MOMP and dmLT suggests that protection is not mediated by cross-reacting antibodies, but may be due to activation of innate immunity. As dmLT is safe for humans, it could be incorporated into a *C*. *jejuni* vaccine to enhance its efficacy.

## Introduction

*Campylobacter jejuni*, a foodborne pathogen, is a major cause of bacterial diarrhea worldwide [1–3]. *C*. *jejuni* infection also results in serious complications such as the Guillain-Barre syndrome, Reiter’s syndrome, reactive arthritis, and irritable bowel syndrome [4]. The economic burden of diarrhea and the complications due to *C*. *jejuni* are considerable [5]. Therefore, many agencies including the World Health Organization have declared the development of a vaccine against *C*. *jejuni* a priority [6, 7]. Whole bacteria and bacterial components have been investigated as possible vaccine candidates, but no candidate has reached a clinical testing stage [8]. We have shown that rabbit polyclonal antibodies to cholera toxin [CT] react with the major outer membrane protein (MOMP) of *C*. *jejuni* [9] and several other species of the *Campylobacter* genus [10]. Furthermore, oral immunization of adult mice with CT afforded significant protection against intestinal colonization with *C*. *jejuni* upon oral challenge [11].

The heat-labile enterotoxin (LT) of enterotoxigenic *Escherichia coli* (ETEC) and CT are functionally and structurally related [12]. LT and its single amino acid mutant, LT (R192G-glycine substituted for arginine in a proteolytically-sensitive site in the A-subunit that separates A1 and A2, preventing cleavage by trypsin) (mLT) have been shown to be strong oral vaccine adjuvants [13–15]. However, the mLT retained reactogenicity and could not be used in humans [16,17]. Therefore, a double-mutant, LT (R192G/L211A-the latter mutation in a putative pepsin-sensitive proteolytic site in the A2 domain, changing leucine 211 to alanine) (dmLT) was subsequently developed [18]. This dmLT retained its adjuvant properties in animal studies [19,20], and was also found to be devoid of reactogenicity for mice [18]. In a recent human volunteer study, oral feeding of dmLT was found to be safe [21]. This has opened up avenues for the use of dmLT as an adjuvant in human vaccination.

In our continuing efforts to develop a vaccine against *C*. *jejuni*, we wished to find out whether dmLT cross-reacts with *C*. *jejuni* and whether oral immunization with dmLT would protect against intestinal colonization with *C*. *jejuni* upon oral challenge of immunized adult mice. We were also interested in ascertaining whether dmLT, being an adjuvant, would afford some degree of nonspecific protection against *C*. *jejuni* even if it did not cross-react with *C*. *jejuni*.

## Materials and Methods

### Ethics statement

This study was carried out in strict accordance with the recommendations of ARRIVE guidelines of the European Union for the care and use of laboratory animals. The protocol was approved by the Committee on the Ethics of Animal Experiments of Kuwait University Faculty of Medicine (Permit Number MI03/13).

The three human *C*. *jejuni* isolates used in this study were isolated from diarrheal stools of patients. These stools were processed at the routine Clinical Microbiology Laboratory of Mubarak Al-Kabir Hospital, Kuwait.

For investigation in the routine clinical laboratory for patient care, no ethical approval is required. Moreover, as these isolates have been used in previous two studies [11,12], the Human Ethics Committee of Kuwait University of Faculty of Medicine waived the requirement for ethics approval for the current study.

### Bacteria and culture conditions

The three isolates of *C*. *jejuni* used in the study were 48 (Penner serotype O:19), 75 (serotype O:3), and 111 (serotype O: in 1,44) which originated from the stools of diarrheic patients as stated above. They were found to colonize mouse intestine in previous studies [11, 22]. Stock cultures were maintained in Brucella broth (Becton & Dickinson, Sparks, MD) with 15% (vol/vol) glycerol at -70°C. Depending upon the purpose, three types of media were used for cultivation. These were: blood agar base (Oxoid, Basingstoke, Hampshire, England) with 5% defibrinated sheep blood; *Campylobacter*-selective agar with laked horse blood, growth supplement and selective supplement (Oxoid); and a biphasic medium with brain heart infusion agar and broth supplemented with 1% yeast extract [23]. Cultures were incubated at 42°C for 48 h in a microaerobic atmosphere generated by Campigen (Oxoid). The identity of the bacteria was confirmed by cultural characteristics and molecular methods. The three serotypes generated unique fingerprints by *flaA* restriction fragment length polymorphism (RFLP) analysis [24].

### Animals

Studies were done on *Campylobacter*-free BALB/c mice (6 to 8 weeks old) obtained from the Animal Resources Center, Health Sciences Center, Kuwait University, and housed separately for 1 week prior to experimentation. The animals were fed a standard laboratory chow (Special Diet Food Services, Ltd., Essex, United Kingdom).

### Preparation of enriched MOMPs

The MOMPs of *C*. *jejuni* 48, 75 and 111 were enriched by the Sarkosyl method [25]. Briefly, the bacteria were grown on blood agar at 42°C for 48 h in a microaerobic atmosphere. Bacterial cells were disrupted by sonication and centrifuged at 5000 X g to remove whole cells. The supernatant was centrifuged at 100, 000 X g for 1 h at 4°C in an L8-70 ultracentrifuge (Beckman, Fullerton, CA). The resultant pellet was then treated with sodium lauryl sarcosinate (Sigma, St. Louis, MO). The Sarkosyl-insoluble portion was used as the enriched MOMP.

### SDS-PAGE of MOMP and Western blotting

The enriched MOMP preparation was separated by discontinuous sodium dodecyl sulfate-polyacrylamide gel electrophoresis (SDS-PAGE) with a 5% stacking gel and a 12.0% separating gel according to the method of Laemmli [26], and stained with Coomassie blue. For Western blotting, the separated proteins were transferred electrophoretically on to a nitrocellulose membrane (Bio-Rad, Hercules, CA) and then blocked with 5% bovine serum albumin (BSA) (Sigma) in phosphate-buffered saline (PBS, pH 7.2) containing 0.05% Tween 20 (PBSTB). The membrane was reacted with an appropriate dilution of relevant rabbit polyclonal antibody in PBST with 1% BSA (PBSTb). The secondary antibody, peroxidase-conjugated, AffiniPure, goat IgG anti-rabbit immunoglobulin, Fc fragment-specific (Jackson ImmunoResearch, West Grove, PA), diluted 1 in 50,000, in PBSTb was added, after which the membrane was developed with enhanced-chemiluminescence Western blotting detection reagents according to the manufacturer’s instructions (Amersham Pharmacia Biotech, Little Chalfont, Buckinghamshire, United Kingdom).

### Production of rabbit antibodies to MOMP of *C*. *jejuni* 111

The Sarkosyl-enriched MOMP from *C*. *jejuni* 111 was separated by SDS-PAGE. The protein band corresponding to the MOMP (~ 45 kDa) was excised from the gel. The band was homogenized in PBS (pH 7.2) and approximately 1 ml of the gel suspension containing 50 μg of the protein was mixed with an equal volume of incomplete Freund’s adjuvant (Sigma) and injected subcutaneously into an adult New Zealand White rabbit. This first dose was followed by two additional doses at 2-week intervals. The rabbit was bled three weeks after the last injection [27].

### Production of rabbit antibodies to dmLT (R192G/L211A)

The dmLT was a gift from John D. Clements, Tulane University Medical Center, New Orleans, LA [18]. A previously used protocol for production of antibody to LT was followed [28]. Briefly, 25 μg of dmLT in PBS (pH 7.2) was emulsified with an equal volume of Freund’s complete adjuvant and injected intramuscularly into an adult New Zealand White rabbit. The rabbit received two booster injections on days 25 and 50 with 25 μg of protein. The first booster dose was emulsified in Freund’s complete adjuvant and the second in incomplete adjuvant. The rabbit was bled three weeks after the second booster dose.

### Rabbit antibodies to LT-EK112K

LT-E112K is a mutant of the LT with a Glu112 → Lys substitution in the A-subunit. Because of this substitution, it is devoid of toxicity for animals, but retains the A and B immunogenic epitopes of native LT [29]. As native LT is highly toxic, sufficient quantities of LT cannot be injected into animals to produce high-titer antisera, but LT-E112K can be used instead. A high titer rabbit antiserum to LT-E112K was a gift from John D. Clements, Tulane University Medical Center.

### Assay for serum antibodies in rabbits

The wells of a Maxisorp immunoplate (Nunc, Rochester, NY) were coated with 100 μl of appropriate antigen (10 μg/ml of *C*. *jejuni* 111 MOMP or 1 μg/ml of dmLT) in carbonate buffer (pH 9.6) at 4°C for 24 h. The wells were washed three times with PBST and blocked with PBSTB at room temperature (RT) for 1 h. Serial doubling dilutions of the rabbit serum (starting at 1 in 200) in PBSTb were added to the wells, followed by incubation at 37°C for 1h. The wells were washed three times with PBST and then incubated at 37°C for 1 h with horseradish peroxidase-conjugated goat anti-rabbit antibody (Jackson ImmunoResearch) diluted to 1 in 1,000 in PBSTb. The wells were washed three times with PBST, after which the substrate, ABTS (2,2’-azino-di-(3-ethyl-benzthiazoline sulfonate) (Sigma) was added. After incubation for 30 min at RT, the optical density (OD) at 405 mm was measured on a PowerWave microplate spectrometer (BioTek Instruments Inc., Potton, Bedfordshire, United Kingdom). End point titers were expressed as the reciprocal of the dilution giving an absorbance value ≥2 standard deviations above the background absorbance in wells containing pooled serum samples from 5 unimmunized rabbits [30].

### Vaccination

Oral vaccination of mice was carried out as described previously [11]. Shortly before vaccination, gastric acidity was neutralized with two 0.5-ml doses of 5% (wt/vol) sodium bicarbonate (pH 8.5) given by oral gavage 15 min apart. Oral administration was done by using a stainless steel, curved-ball-tip feeding needle (20 gauge, 1.5” long) (Popper & Sons Inc., New Hyde Park, NY). Mice were vaccinated three times at weekly intervals. Each dose consisted of 25 μg of dmLT in 300 μl of PBS (pH 7.2). Control mice received PBS (pH 7.2). There were 12 mice in the test group and an equal number in the control group. Animals were monitored daily for signs of illness, including lethargy, refusal to feed, loss of weight, ruffled fur and diarrhea for the duration of the study. A veterinarian assisted with these assessments. All animals remained well till the end of the study.

### Challenge of vaccinated mice

Mice were orally challenged with bacterial cultures, *C*. *jejuni* 48 and 111 separately, a week after the third vaccine dose, and immediately after the collection of feces and blood. Before challenge, gastric acidity was neutralized with sodium bicarbonate. Then, a 0.5-ml suspension containing 1 X 10^9^ CFU per ml of a challenge strain in PBS (pH 7.2) was given orally. The suspensions were prepared from bacteria grown in biphasic medium overnight. Mice were monitored daily for signs of illness as described above, and for shedding of the challenge strain in their feces. Fresh fecal pellets from each mouse (100 to 120 mg) were collected and diluted to 10% in PBS (pH 7.2), followed by 5-fold serial dilutions. An aliquot of 50 μl of each dilution was plated in duplicate on *Campylobacter*-selective agar plates. After 48 h, *C*. *jejuni* colonies were counted and expressed as the number of CFU per mg of feces. Strain identity was confirmed by *flaA* RFLP analysis when required. Mice were considered not colonized if their feces yielded no *Campylobacter* colonies (detection limit = 200 CFU per g). Vaccine efficacy was calculated as the (rate for control mice-rate for vaccinated mice)/rate for control mice) X 100, where the rate is % of mice colonized [23]. At the end of the study, mice were euthanized with inhaled carbon dioxide.

### Collection of feces and blood for antibody estimation

Five to seven fresh fecal pellets (equivalent to 100 to 200 mg) were collected from each mouse shortly before vaccination and a week after the third vaccine dose. This time schedule showed a good immune response to CT in our previous study [11]. To each mg of the feces, 100 μl of IgA extraction buffer (PBS containing 0.05% Tween 20, 0.5% fetal calf serum, 1 mg/ml ethylenediaminetetraactetic acid [EDTA], 1 mg/ml phenylmethylsulfonyl fluoride, and 200 μg/ml trypsin soybean inhibitor) was added. After 15 min of incubation on ice, fecal pellets were homogenized and centrifuged at 23,000 X g for 15 min at 4°C. The supernatant was stored at -70°C until assayed [31].

At the same times as the collection of feces, tail vein blood was collected. 50 μl of blood were mixed with 950 μl of PBS (pH 7.2) containing 0.1% Tween 20. After one cycle of freezing and thawing, the sample was centrifuged at 400 X g for 15 min, and the supernatant was stored was at -70°C until assayed [30].

### Measurement of total IgA in fecal extract

The total IgA in fecal pellets was determined by using an ELISA [31]. The wells of a Maxisorp immunoplate (Nunc) were coated with affinity purified, human serum absorbed, goat anti-mouse IgA (KPL, Gaithersburg, MD) in carbonate buffer and incubated at 37°C for 1 h followed by 24 h at 4°C. The wells were washed three times with PBST and blocked with PBSTB at 37°C for 2 h. Various concentrations (3 to 300 ng/ml) of purified mouse IgA (Bethyl Laboratories, Montgomery, TX) or serial doubling dilutions (starting from 1 in 100) of fecal extracts (from pre-immunized and post-immunized mice) in PBSTb were added to the wells and incubated at 37°C for 2 h. The wells were then washed three times with PBST and then incubated with horseradish peroxidase-conjugated, goat anti-mouse IgA antibody (Sigma)diluted to 1 in 5,000 in PBSTb at 37°C for 2 h. Wells were washed three times with PBST, after which ABTS was added. After 30 min at 37°C, the OD_405 nm_ was measured. The amount of IgA present in a fecal sample was determined by interpolation of OD on a standard curve constructed with OD readings of known concentrations of purified mouse IgA.

### Measurement of dmLT antibodies in feces

The dmLT-specific IgA antibody was measured in fecal pellets by using an ELISA. The wells of a Maxisorp immunoplate (Nunc) were coated with 1 μg/ml dmLT in carbonate buffer at 4°C for 24 h. Wells were washed three times with PBST and blocked with PBSTB at RT for 1 h. Serial doubling dilutions (starting from 1 in 20) of fecal extracts in PBSTb were added to the wells and incubated at RT for 1 h. The wells were washed three times with PBST and then incubated with horseradish peroxidase-conjugated goat anti-mouse IgA antibody (Sigma) diluted 1 in 5,000 in PBSTb at RT for 1 h. The addition of substrate, incubation and OD readings were done as described above. Endpoint titers were expressed as the reciprocal of the highest dilution giving an absorbance value ≥2 standard deviations above the background absorbance in wells containing fecal extract from unimmunized mice [29]. The reciprocal specific IgA antibody titer was divided by the total IgA concentration (in μg/mg) in the feces of each mouse to adjust for variation between mice [32].

### Measurement of dmLT antibodies in blood

dmLT-specific antibodies (IgG or IgA isotype) were measured in blood samples by using an ELISA [30]. dmLT-coated wells of a Maxisorp immunoplate (Nunc) were reacted with serial doubling dilutions of lysed blood samples (starting with a 1 in 200 dilution) in PBSTb. After washing, the wells were reacted with horseradish peroxidase-conjugated goat antibody to mouse IgA (1 in 5000 dilution in PBSTb)or IgG (diluted to 1 in 1,000) (both Sigma). OD readings were taken after reaction with the substrate, and endpoint titers were expressed as the reciprocal of the highest dilution giving an absorbance value of ≥2 standard deviations above the background absorbance in wells containing lysed-blood samples from unimmunized mice.

### Measurement of MOMP IgA antibodies in feces

The ELISA procedure was similar to that used to measure anti-dmLT antibodies in feces. The antigen used was the enriched MOMP from *C*. *jejuni* 111 and a single dilution (1 in 20) of fecal extract was tested.

### Measurement of MOMP antibodies in blood

Wells of a Maxisorp immunoplate (Nunc)were coated with enriched MOMP from *C*. *jejuni* 111. A single 1 in 100 dilution of lysed blood was tested. The secondary antibodies used were goat anti-mouse IgG and IgA in separate assays with endpoint antibody titers determined as for dmLT antibodies.

### Statistical analysis

Statistical analysis was done by using SPSS version 17 (SPSS, Chicago, IL). Comparison between rates of colonization of mice was made by using Fisher’s exact test. Comparisons of endpoint antibody titers and fecal *C*. *jejuni* colony counts were made by using the Mann-Whitney test. Values differing with a *P* value of ≤0.05 were considered statistically significant [11, 22].

## Results

### Cross-reactivity of MOMPs from *C*. *jejuni* 48, 75 and 111

**Fig 1:**
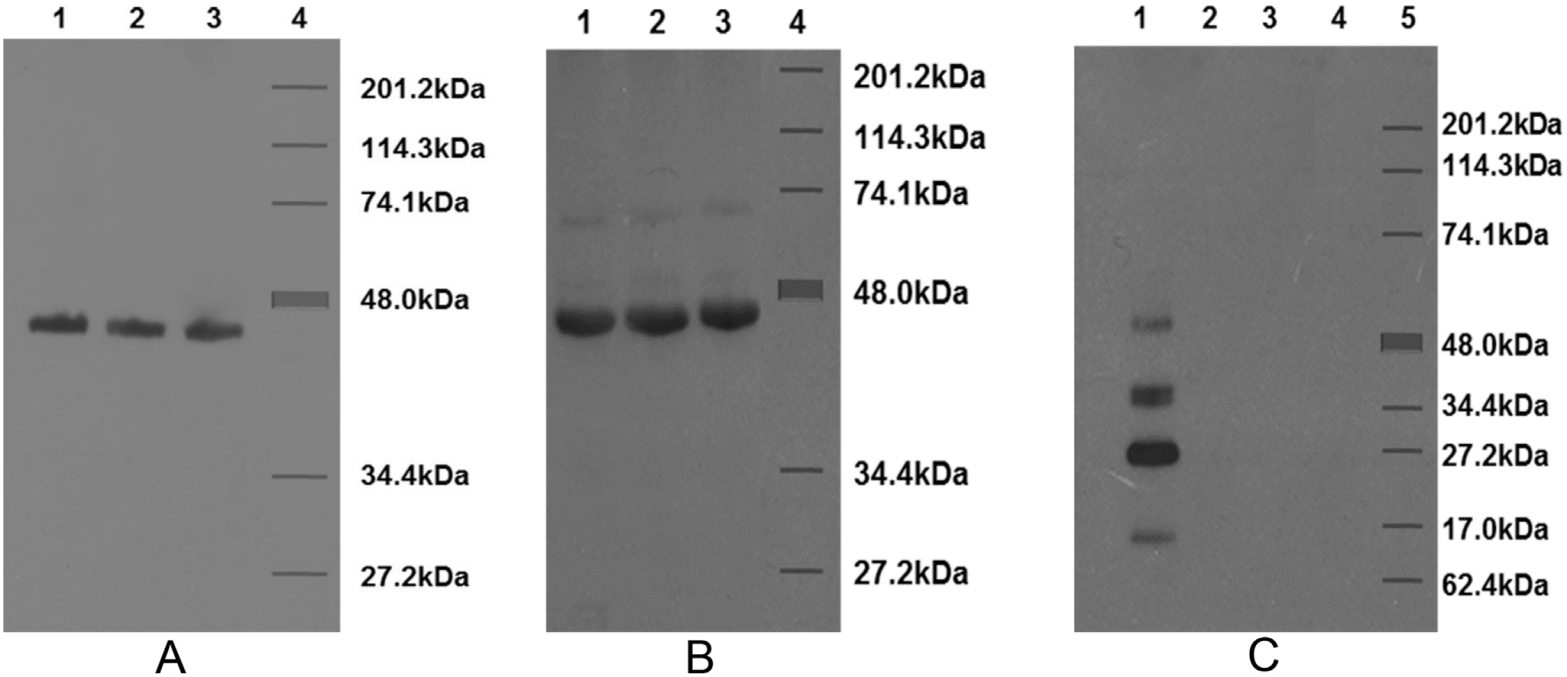
Western blots of enriched MOMPs from *C*. *jejuni* strains reacted with rabbit antiserum to the MOMP from *C*. *jejuni* strain 111 (A), with rabbit antibody to CT (Sigma) (B), and with rabbit antiserum to dmLT (C). SDS-12% PAGE-separated MOMPs were transferred to a nitrocellulose membrane and probed with the appropriate rabbit antiserum and then with anti-rabbit immunoglobulin. In panels A and B, lanes 1, 2 and 3 were loaded with MOMPs from *C*. *jejuni* 48, 75 and 111, respectively; lane 4 contains molecular weight markers. A prominent band of approximately 48 kDa corresponding to the MOMP is seen in all the three strains in panels A and B. The light band of approximately 74.1 kDa in panel B is a nonspecific band [9]. In panel C, lane 1 was loaded with dmLT, and 2 through 5, were the same as lanes 1 through 4 in panels A and B. There is a lack of reaction between the MOMPs and dmLT antibody in panel C. The different bands in lane 1 may represent different components of dmLT, namely B_5_, A, A1 and B_1_ (from highest to lowest molecular weight).

The homologous titer of rabbit antibodies to the MOMP of *C*. *jejuni* 111 in ELISA was 1 in 128,000. This antibody (at 1 in 10,000 dilution) reacted strongly on Western blot with all three strains (Fig 1A). This shows the sharing of MOMP antigens among the *C*. *jejuni* strains used for this study.

### Cross-reactivity of CT antibodies with *C*. *jejuni* 48, 75 and 111

We have previously shown that anti-CT antibodies react with MOMPs from various strains of *C*. *jejuni* [9]. This was confirmed in the present study as CT antibody (Sigma, at 1 in 2000 dilution) reacted with the MOMPs from all three strains of *C*. *jejuni* (Fig 1B).

### Lack of reactivity of anti-dmLT antibodies and anti-LT-E112K antibodies with *C*. *jejuni* 48, 75 and 111

The titer of rabbit antibody to dmLT in ELISA was > 1 in 128,000. We tested various dilutions of this antibody (1 in 1000, 1 in 2000, 1 in 5000, 1 in 10,000) on Western blot against MOMPs from the three strains of *C*. *jejuni*. At all dilutions tested, the antibody reacted with dmLT, but not with the MOMPs. A sample reaction is shown in Fig 1C.

The Western blot was repeated with various dilutions of LT-E112K antibodies, but did not show any reaction with the MOMPs from the three *C*. *jejuni* strains. However, the antibodies did react with the dmLT antigen as above (data not shown).

### Colonization of *C*. *jejuni* strains and vaccine efficacies

Vaccination and challenge studies were carried out with *C*. *jejuni* strains 48 and 111. Mice did not show any signs of illness after vaccination or after bacterial challenge.

**Fig 2:**
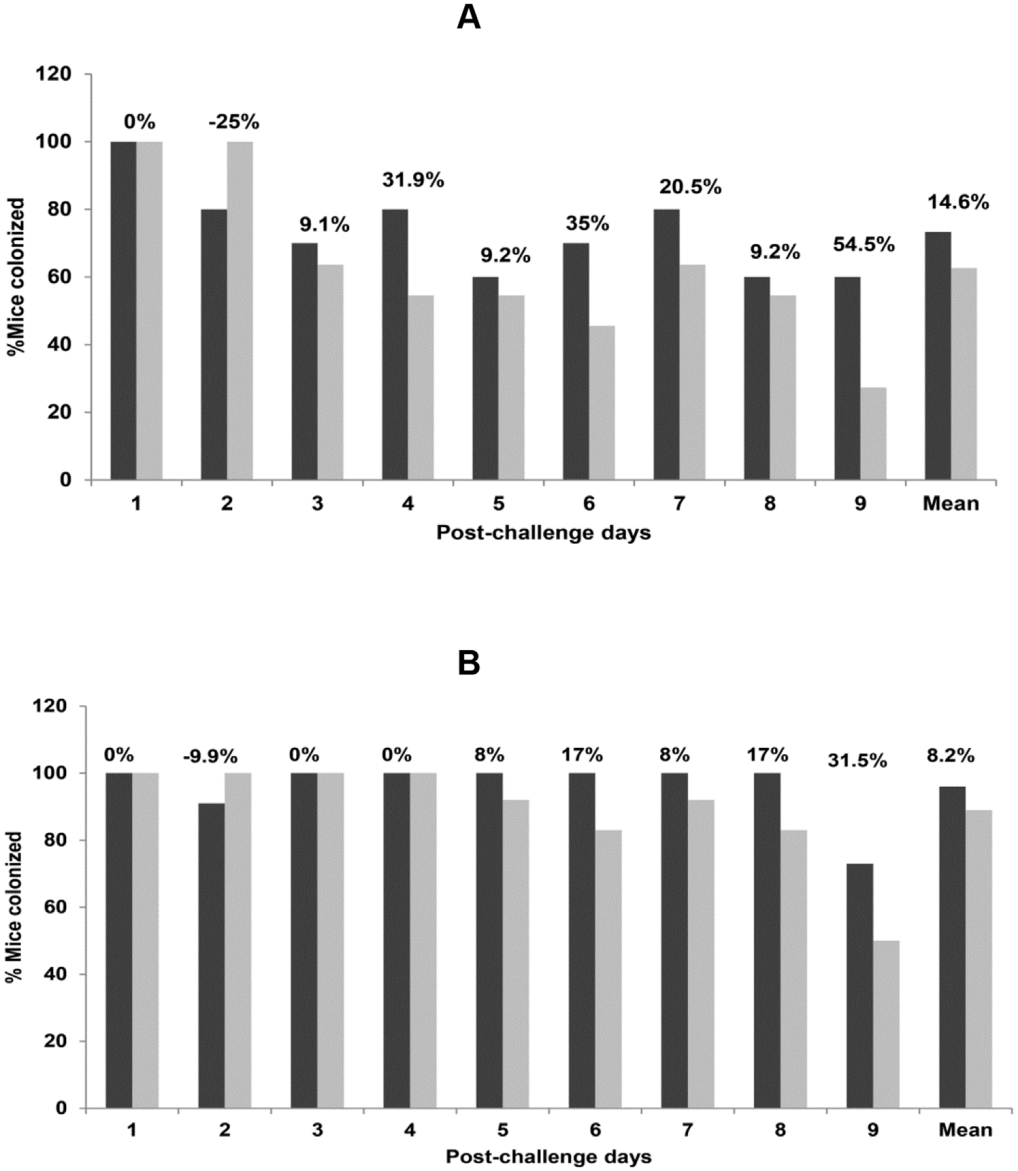
Colonization of mice by *C*. *jejuni* 48 (A) and by *C*. *jejuni* 111 (B). The X-axes represent post-challenge days and the Y-axes indicate % colonization. “Mean” is the mean value for all 9 days. No statistical difference in colonization was seen between the dmLT-vaccinated (light bar) and control (dark bar) mice for both strains. The protective efficacy of the vaccine for each day is shown above the corresponding bars.

The colonization rates of vaccinated mice for 7 of 9 days and the mean bacterial counts in feces were lower in vaccinated than in control mice for strain 48. The protective efficacy of the dmLT vaccine varied from 9.1% to 54.5% on these 7 days. However, none of the comparisons for colonization between the vaccinated and control mice were statistically significant (Fig 2A).

For strain 111, the colonization rates for the last 5 days and the mean colonization were lower in vaccinated mice, and the protective efficacy of the vaccine varied from 8% to 31.5% on these days. Again, none of the comparisons for colonization between the vaccinated and control mice were statistically significant (Fig 2B).

### Fecal excretion of *C*. *jejuni* strains

**Fig 3:**
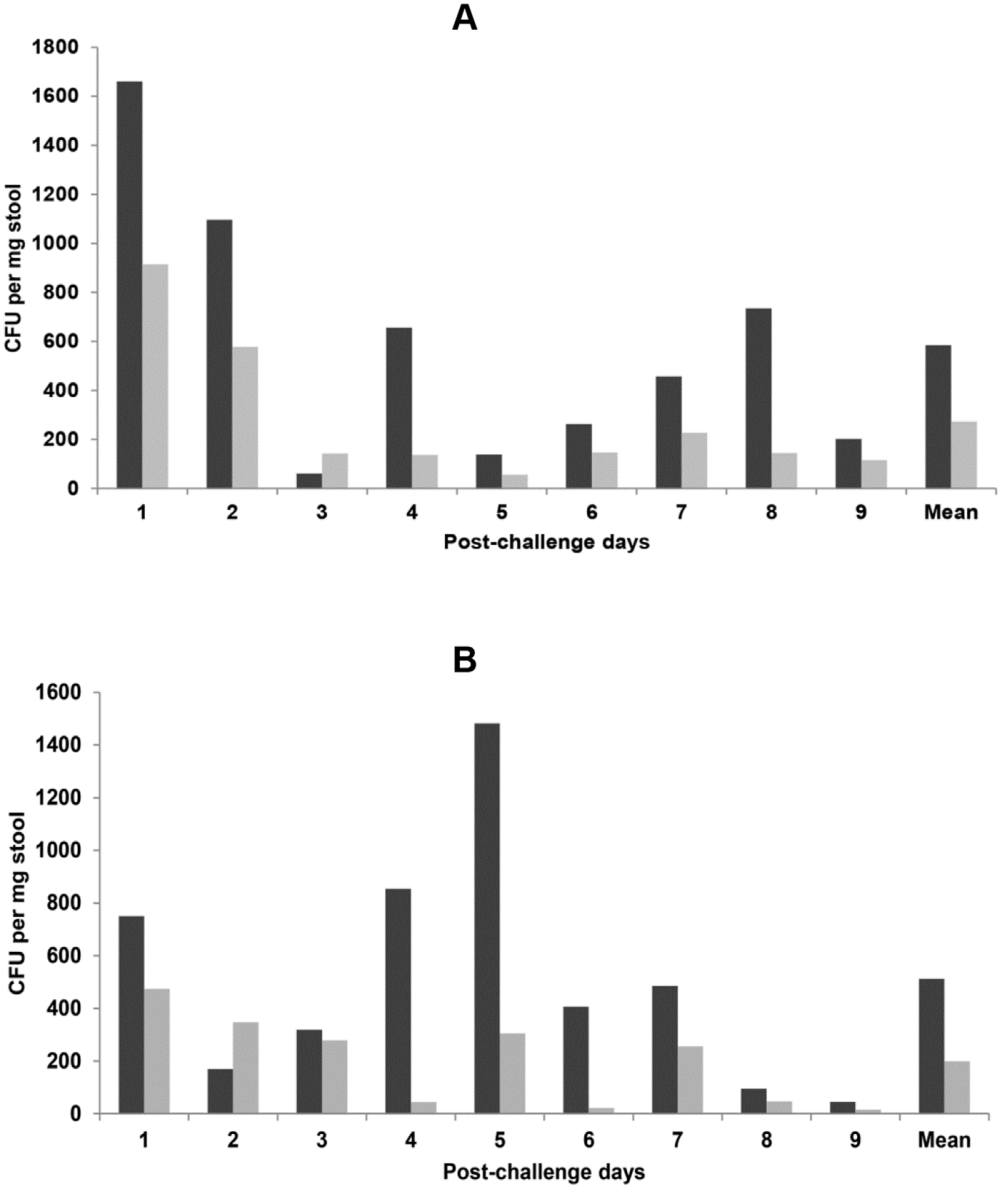
Fecal excretion by mice of *C*. *jejuni* 48 (A) and *C*. *jejuni* 111 (B). The X-axes represent post-challenge days and the Y-axes show the number of bacteria excreted per mg feces. “Mean” is the mean for all 9 days. Reduced excretion by the vaccinated mice (light bar) compared with the control mice (dark bar) was not statistically significant for any day or mean excretion for *C*. *jejuni* 48, but significant for days 4 and 6 (*P* = 0.008 for both days) for *C*. *jejuni* 111.

There was a reduced excretion of *C*. *jejuni* 48 by vaccinated mice on all nine days, but the differences were not statistically significant (Fig 3A). A similar pattern of reduced excretion by vaccinated mice was observed for *C*. *jejuni* 111 attaining statistical significance on days 4 and 6 (Fig 3B).

### Antibody responses to dmLT and *C*. *jejuni* MOMP

A total of 24 mice were vaccinated with dmLT before challenge with *C*. *jejuni* 48 and 111. Blood and stool from every other mouse (from a total of 12 mice)were tested for antibody response (Table 1). Pre-vaccination blood samples contained no detectable IgG antibodies to dmLT, but showed a low titer of IgA antibodies to this antigen. Post-vaccination serum samples contained IgG antibodies and a significantly increased titer of IgA antibodies (P <0.001), and the rise in IgA antibody titer was statistically significant. The increase in fecal IgA antibodies to dmLT was also statistically significant (P <0.001).

**Table 1:** Serum and stool antibody levels* to dmLT in immunized and control mice.

Immunized mice			Control mice		
Serum IgG	Serum IgA	Stool IgA	Serum IgG	Serum IgA	Stool IgA
4.99 ± 0.39**	4.74 ± 0.53**	3.67 ± 0.16**	0	2.28 ± 0.73	2.13 ± 0.24

*Log_10_ titer ± standard deviation

**Significantly greater than control mice (P <0.001)

Neither the pre-vaccination nor the post-vaccination samples contained detectable antibodies to the MOMP from *C*. *jejuni* 111 at the dilution tested.

## Discussion

Epidemiological studies indicate that immunity develops following *C*. *jejuni* infection and that this is correlated with the development of antibodies. Children <2 y of age in developing countries develop a less severe disease compared to their counterparts in developed countries. This partial protection is mediated by maternally-derived antibodies and early exposure to the pathogen [33– 35]. Also, children experience a progressive increase in titers of all isotypes of *C*. *jejuni*-specific serum antibodies in the first two years of life, followed by an ongoing increase in IgA titers, indicative of frequent exposure to the organism and a boost to mucosal immunity [34]. In industrialized countries, habitual raw milk-drinkers have a reduced incidence of *C*. *jejuni*-specific diarrhea and high levels of specific serum antibody compared to first time drinkers [36]. In volunteer studies, short-term protection from illness upon re-challenge with homologous bacterial strain was correlated with elevated titers of serum and intestinal antibodies to the organism [37]. Together, these data suggest that a *Campylobacter* vaccine which evokes a systemic and mucosal antibody responses is likely to afford some protection against infection. Because we have previously shown that mucosal immunization with CT can evoke a specific anti-*Campylobacter* antibody response (11), we investigated the ability of dmLT, a candidate mucosal adjuvant closely related to CT, could do the same.

Our results showed that mice given dmLT did not exhibit any clinical signs of illness after the administration of dmLT. This was not unexpected given that dmLT has been shown to be devoid of toxicity in animal [18] and human [21] studies. Mice also remained well after inoculation with *C*. *jejuni*, because *C*. *jejuni* does not induce signs of sickness in the adult mouse intestinal colonization model [22].

The MOMP of *C*. *jejuni* is a major surface protein, which can mediate attachment to the epithelial cell surface [38]. This MOMP possesses unique and cross-reacting epitopes with MOMPs from different serotypes of *C*. *jejuni* [27]. In the current study, we showed that antibody to the MOMP of *C*. *jejuni* 111 cross-reacted with the MOMPs from two unrelated strains. The sera of patients recovering from *C*. *jejuni* infection [39,40], as well as those of animals immunized with *C*. *jejuni* [41], possess antibodies to MOMPs, and a MOMP vaccine provided heterologous protection in an animal model of infection [22].

Oral immunization of adult mice with CT provided significant protection against colonization with different serotypes of *C*. *jejuni*. As CT and MOMP evidently share some epitope(s), the cross-reacting antibodies are assumed to mediate protection against *C*. *jejuni* in mice immunized with CT [11]. However, antibodies to dmLT did not cross-react with the MOMPs from *C*. *jejuni* strains on Western blot. This finding was corroborated by the lack of detectable serum and fecal antibody responses to the MOMP by ELISA, in mice immunized dmLT, notwithstanding a robust antibody response to dmLT itself. We also investigated whether antibodies to the native LT homologue, LT-E112K, reacted with the MOMPs, but there was no reaction either. The different results seen with CT and derivatives of LT are likely to be due to the fine differences that exist between CT and LT throughout their structure [42]. Therefore, the epitope(s) present in CT responsible for the cross-reaction with the *C*. *jejuni* MOMP, may be absent in dmLT and LT-E112K. It was not appropriate to test LT as a control in vaccination studies, because of the lack of cross-reaction with it. Moreover, it is toxic to mice. Similarly including *C*. *jejuni* MOMPs as a control antigen in vaccination studies is not appropriate, as we have shown previously that MOMP with mLT as an adjuvant is a good subunit vaccine which imparted significant protection in the mouse colonization model of infection [22].

In accordance with the lack of cross-reaction, the level of protection afforded by immunization with dmLT was not significant, unlike when mice were immunized with CT which imparted significant protection against *C*. *jejuni* [11]. However, in spite of the lack of cross-reaction between MOMP and dmLT, some protection (albeit not statistically significant) was observed against colonization. Specifically, the number of *C*. *jejuni* recovered from the feces of dmLT-immunized mice was consistently less that in control mice, and this even achieved statistical significance for *C*. *jejuni* strain 48 on two of nine days of the follow-up. This suggests that dmLT offered some degree of nonspecific protection against *C*. *jejuni* colonization.

Although adjuvants themselves do not normally impart specific protection to a pathogen, we found that dmLT provided specific partial protection to infection with *C*. *jejuni*. The mechanisms by which this was mediated are not known and will require further investigation to elucidate.

Even the mechanisms of adjuvanticity of LT and its derivatives, mLT and dmLT, are mostly unknown. However, when peripheral blood leukocytes from individuals exposed to vaccines or diseases are stimulated with specific antigens, they show enhanced IL-17A production, which in turn may act by enhancing antibody production and T cell responses. The IL-17A potentiating effect of dmLT is partly exerted via antigen presenting cells (APCs) and partly via recruitment and activation of neutrophils and macrophages (summarized in [43]). In fact, studies in mice suggest that LT can promote protective immune responses in part by inducing innate IL-1 and IL-23, thus stimulating Th17 cells [44]. In an ex-vivo model of infection with human intestinal biopsies, inoculation with *C*. *jejuni* induced IL-17A production which reduced the number of intracellular *C*. *jejuni* in the intestinal epithelium, suggesting that this cytokine may contribute to protective immunity against this bacterium [45].

The results of our study suggest that dmLT is likelt to be a useful adjuvant for incorporation into a *C*. *jejuni* vaccine. Indeed, experimental *C*. *jejuni* vaccines have already shown the potentiating effect of LT in animal studies [14], and of mLT in human studies [17].

## Conclusion

In this animal study, we have shown that immunization with the mucosal adjuvant, the double mutant heat-labile enterotoxin of *E*. *coli*, LT (R192G/L211A) (dmLT) imparts partial protection against *C*. *jejuni* colonization and excretion, and that this protection may be mediated by enhancement of innate immunity. Incorporation of dmLT as a mucosal adjuvant should improve the efficacy of a human *C*. *jejuni* vaccine.
